# Supplementary material for: A Comprehensive Assessment of Ultraviolet-Radiation-Induced Mutations in Flammulina filiformis Using Whole-Genome Resequencing
Source: J Fungi (Basel). 2024 Mar 20;10(3):228. doi: 10.3390/jof10030228 (PMC10971301; doi:10.3390/jof10030228)
Supplement: Supplementary file 1 [file jof-10-00228-s001.zip › Supplementary Material S8/KEGG annotation/out/64381550635650.os/KO/out_map/map03060.html]

KEGG PATHWAY: Protein export - Reference pathway


|  |  |
| --- | --- |
| **Protein export - Reference pathway** |  |

[
Pathway menu
| Organism menu
| Pathway entry
| Show description
| User data mapping
]

The protein export is the active transport of proteins from the cytoplasm to the exterior of the cell, or to the periplasmic compartment in Gram-negative bacteria. The sec dependent pathway is the general protein export system that transports newly synthesized proteins into or across the cell membrane. The translocation channel is formed from a conserved trimeric membrane protein complex, called the Sec61/SecY complex. The twin-arginine translocation (Tat) pathway is another protein transport system that transports folded proteins in bacteria, archaea, and chloroplasts. Many Tat systems comprise three functionally different membrane proteins, TatA, TatB, and TatC, but TatA and TatE seem to have overlapping functions, with TatA having by far the more important role.

|  |  |
| --- | --- |
| Reference pathway | 100% |
